# Current Approach for Diagnosis and Treatment of Adrenal Tuberculosis—Our Experience and Review of Literature

**DOI:** 10.1055/s-0042-1743523

**Published:** 2022-03-03

**Authors:** Stuti Gupta, Md Abu Masud Ansari, Arun Kumar Gupta, Poras Chaudhary, Lalit Kumar Bansal

**Affiliations:** 1 Department of Microbiology, All India Institute of Medical Sciences, New Delhi, India; 2 Department of Surgery, Atal Bihari Vajpayee Institute of Medical Sciences, Dr. Ram Manohar Lohia Hospital, New Delhi, India

**Keywords:** Addison's disease, adrenal tuberculosis, antituberculous therapy, adrenal insufficiency

## Abstract

Addison's disease was first described by Thomas Addison in 1855. He demonstrated the destruction of bilateral adrenal gland by tuberculosis (TB) in six patients. Since then, the incidence of TB has declined in the Western world, but in developing countries, it is still the most common cause of adrenal insufficiency. Because of the introduction of antituberculous chemotherapy, the incidence of adrenal TB has been declined in the past decades. The most common symptoms are nonspecific; therefore, diagnosis is often delayed, and patients may first present with a life-threatening adrenal crisis. The most commonly identified organism for adrenal failure in adrenal TB is *Mycobacterium tuberculosis* infection. Adrenal TB involves bilateral adrenal glands more frequently than unilateral glands. Computed tomography (CT) scan and magnetic resonance imaging (MRI) are useful investigations to differentiate between tuberculous Addison's disease and the other causes of adrenal insufficiency. In CT scans or MRI, features of adrenal TB are bilateral adrenal enlargement and peripheral rim enhancement with or without calcifications. Antituberculous drugs, biochemical monitoring of adrenal function, and steroid therapy are essential for the management of adrenal TB and adrenal insufficiency. Here, we describe a case of adrenal TB with abscess formation followed by a review of the current literature of adrenal TB for better diagnosis and management of this condition.

Adrenal tuberculosis (TB) is almost always secondary to TB elsewhere, most often from pulmonary and genitourinary TBs. 1 Primary adrenal TB is an infrequent clinical entity, and only a few cases are reported in the literature. In a systematic review, studied by Edlin in 1978, only one case of primary adrenal TB out of 370 patients of extrapulmonary TB was observed during a period of 10 years. 2 TB may affect any of the endocrine glands, including the adrenal, hypothalamus, pituitary, thyroid, and pancreas, but the most commonly involved organ among these is the adrenal gland. 3 In addition to TB, other mycobacteria, bacteria, viruses, and fungus may affect the adrenal glands and may lead to the development of adrenal insufficiency. 3 Most cases of adrenal TB are found 10 to 15 years after the initial infection; hence, tuberculous Addison's disease has a relatively late onset. 4 Addison's disease is defined as primary adrenal insufficiency in which the adrenal gland is not able to produce enough steroid hormones. Addison's disease was first described by Thomas Addison in 1855. 4 He demonstrated the destruction of the bilateral adrenal gland by TB in six patients. 3 4 Since then, the incidence of TB has declined in the Western world, but in developing countries, it is still the most common cause of adrenal insufficiency. 5 In the autopsy series, adrenal involvement is found in ∼6% of patients with active TB. 6 To develop adrenal insufficiency, in adrenal TB, more than 90% of the gland must be destroyed. 7 TB spreads by a hematogenous route to the adrenal glands.

In most cases, adrenal TB is secondary to genitourinary TB or other pulmonary or extrapulmonary TB, or even more rarely primary due to reactivation of the disease. 8 The clinical signs and symptoms of isolated adrenal involvement in TB are nonspecific, such as fever, malaise, anorexia, and abdominal pain. Signs of adrenal insufficiency are very late and only appear after the destruction of at least 90% of the adrenal gland. 7 Ultrasound shows unilateral or bilateral adrenal mass. Computed tomography (CT) scan is more sensitive. In the acute and active phase, CT scan shows adrenal hypertrophy, mostly bilateral, while in chronic cases, CT scan shows peripheral ring enhancement and calcification. 9 Magnetic resonance imaging (MRI) is a better modality than a CT scan because it can identify caseous necrosis, granuloma formation, and other features of active or chronic disease, but a CT scan is more sensitive to detecting calcification. Antituberculous therapy is the mainstay of treatment. A hormone replacement therapy (glucocorticoid and mineralocorticoids) is administered in case of adrenal insufficiency.

## Case Report

**Fig. 1:**
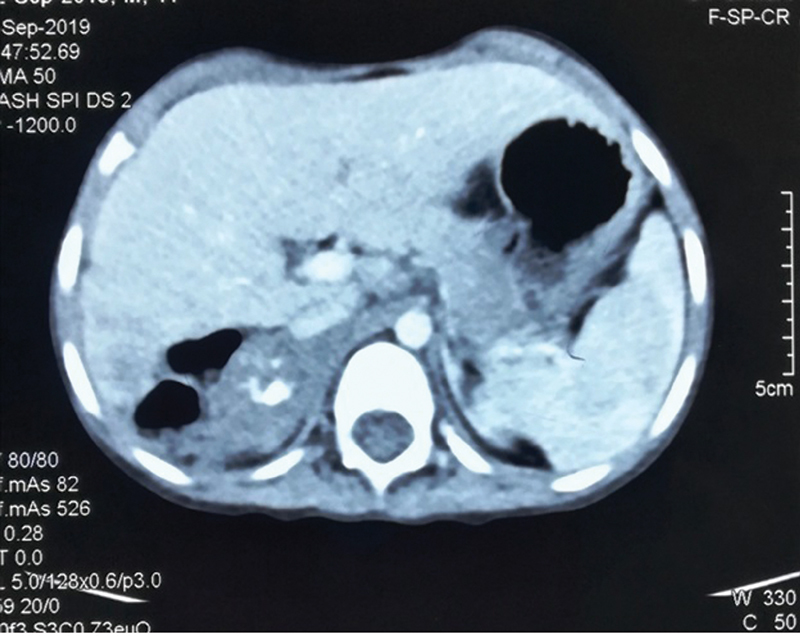
Right side adrenal abscess with calcification (coronal view).

**Fig. 2:**
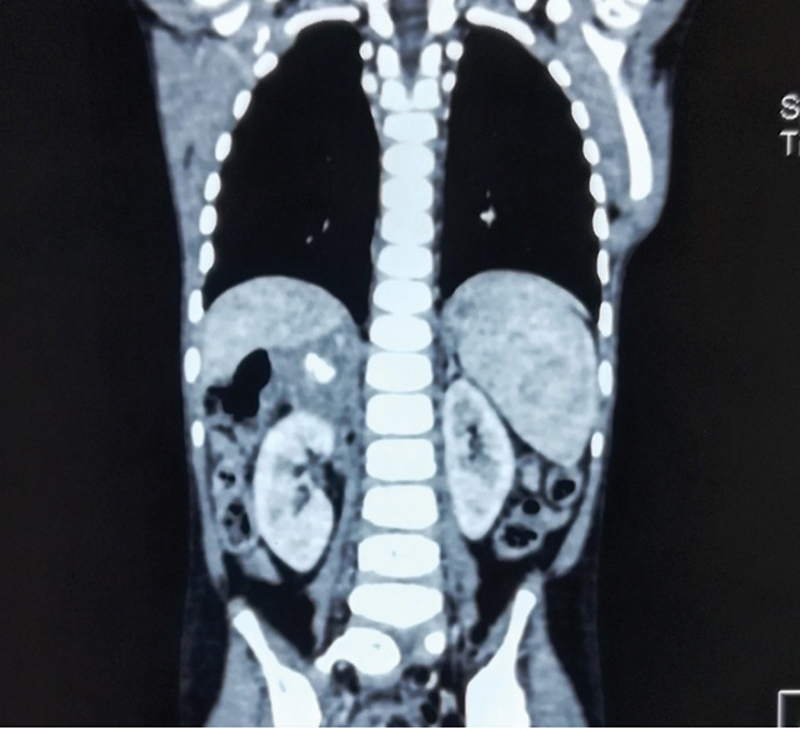
Right side adrenal abscess with calcification (sagittal view).

In our case, a 26-year-old man was admitted in a surgical emergency with fever, abdominal pain with shock. Later, on an investigation, he has been found bilateral adrenal enlargement with abscess formation and calcification on the right side ( Figs. 1 and 2 ). Laboratory features are suggestive of adrenal insufficiency. The patient was not responded to conservative management and went into sepsis. Later right-side adrenalectomy was performed, and the patient condition was improved slowly. On histopathological examination, diagnosis of adrenal TB was confirmed. The patient was discharged on the 13th postoperative day on antitubercular therapy.

## Materials and Methods

An electronic search was undertaken in MEDLINE and PubMed using the terms “adrenal” in combination with “tuberculosis.” A total of 27 studies were identified in various case reports and series. All available articles are reviewed. All resulting titles, abstract, and full text, when available, were read and kept for reference. Clinical signs and symptoms from various studies are summarized in tabulated form. Pathogenesis, clinical features, diagnosis, and treatment were summarized and concluded. Also, we have added a case of adrenal TB admitted in a surgical emergency who is treated later with adrenalectomy.

## Demographic Features

Primary adrenal insufficiency is a rare disorder with a prevalence estimated at ∼120 per million of the population. 10 The most common cause of adrenal insufficiency in developed countries is autoimmune destruction, which is found in ∼75 to 80% of cases. 11 Adrenal TB accounts for ∼7 to 20% of cases. 11 Other rare conditions include bacterial and viral infections, adrenal hemorrhage, histoplasmosis, blastomycosis, adrenal neoplasm or metastatic tumors, and a variety of opportunistic infections due to acquired immunodeficiency syndrome (AIDS). 11 Age and sex are also considered predisposing factors. Males and elderly patients are more likely to have TB infections. 11 The incidence of TB has declined in the Western world, but in developing countries, it is still the most common cause of adrenal insufficiency. 5 10 Because of the introduction of antituberculous chemotherapy, the incidence of adrenal TB has been declined in the past decades. Guttman, in 1930, in his study on 566 cases reported that ∼70% of cases of Addison's disease were caused by TB. 12

## Etiopathogenesis

Addison's disease can occur due to various causes, including various bacterial and viral infections, autoimmune diseases, tumors, and vascular diseases. The most commonly identified organism for adrenal failure is *Mycobacterium tuberculosis* infection. *Mycobacterium tuberculosis* infection spreads to the adrenal glands via a hematogenous route. Most of the patients with active disease have bilateral adrenal enlargement. 7 The explanation of bilateral involvement is that the hematogenous and lymphatic spread from the primary mycobacterial infection reaches both of the adrenal glands with an equal chance. 13 Wang et al reported in their study that all cases of the adrenal glands with bilateral involvement were due to TB. 14 Guo et al, in their study, reported that the occurrence of bilateral involvement was 91% in adrenal TB. 13 After the recovery or in the chronic phase, the enlarged tuberculous adrenal glands return to their normal size because of fibrosis and calcification in the glands. 13 14 Calcification can be diffuse, localized, or punctuated and increases with the duration and course of the disease. 15 In different case series, incidence of calcification is reported from 40 to 59%. 14 16 17 Other conditions that may be found with adrenal calcification are adrenal hemorrhage, histoplasmosis, and blastomycosis. 13 If the condition persists for longer than 10 years, bilateral adrenal glands may become smaller or atrophic. 13

## Clinical Features

The clinical presentation of adrenal TB is nonspecific. Most of the patients with adrenal TB are asymptomatic. Symptoms appear when there is significant destruction of the bilateral adrenal gland. Signs and symptoms of adrenal insufficiency with nonspecific features of TB may be found simultaneously. The most common features are fever, weakness, fatigue, nausea, vomiting, anorexia, weight loss, hypotension, and skin hyperpigmentation. 18

Signs and symptoms of adrenal insufficiency in various studies are described in Table 1 .

**Table 1:** Sign and symptoms of adrenal tuberculosis

Signs and symptoms	Prevalence (%)
Anorexia	75–100
Fever	72–94
Weakness	72–100
Fatigue	70–100
Hyperpigmentation	65–94
Gastrointestinal symptoms (nausea, vomiting, abdominal pain, constipation, and diarrhea)	58–92
Hypotension (systolic blood pressure < 110 mm Hg)	50–90
Salt cravings	5–16
Giddiness	4–12
Vitiligo	10–20
Muscle or joint pain	5–10

## Differential Diagnosis

Other conditions of bilateral adrenal enlargement or insufficiency should be differentiated from adrenal TB. It includes autoimmune adrenalitis, adrenal metastasis, non-Hodgkin's lymphoma, adrenal hemorrhage, histoplasmosis, hypertrophic adrenal changes, and primary adrenal tumors.

Autoimmune adrenalitis is responsible for ∼75 to 80% of Addison's disease in developed countries. 11 Identification of adrenal antibodies in plasma can differentiate it from TB.

Non-Hodgkin's lymphoma was found more commonly than Hodgkin's disease in the adrenal gland. MRI scanning at multiple plane levels can detect extra-adrenal infiltration from lymphoma, especially if the condition is associated with inferior vena cava involvement. 19 20

Adrenal metastases are found bilaterally in most cases, but adrenal metastases rarely cause Addison's disease. The reason behind this is that the residual cortex is likely able to maintain adrenal function. 21

Bilateral adrenal pheochromocytoma should be ruled out in any case of adrenal enlargement. Laboratory tests are useful to discriminate pheochromocytoma from TB. Also, specific features on MRI such as specific bright T2 signals and bright rim-unlike enhancement can distinguish it from adrenal TB. 22

## Diagnostic Tools

Isolation of tubercular bacilli from the adrenal gland is difficult. Isolation may be achieved from secondary locations such as urine in urogenital TB and the sputum in pulmonary TB. It is essential to eliminate a pheochromocytoma before proceeding with any invasive diagnostic and therapeutic procedures. 23 Early identification of people with a high probability of having active TB (presumptive TB) is the most important, and all efforts should be undertaken to microbiologically confirm the diagnosis of presumptive TB cases. Currently, there are three main validated methods for the diagnosis of active TB: microscopy, nucleic acid amplification tests (NAATs), and cultures. 24 25 In addition, antigen detection tests are also available commercially.

### Microscopy

Smear microscopy remains the basis for the diagnosis of TB in developing countries. Two methods of microscopy are currently being used—conventional microscopy using Ziehl–Neelsen's staining or Kinyoun's staining where acid-fast organisms can be visualized on microscopic examination of smears prepared from sputum or other biological specimens and light-emitting diode–based fluorescent microscopy (LED FM) using auramine a fluorescent dye. LED FM is 10 times more sensitive than Ziehl–Neelsen's staining. 24 The limitation of smear microscopy is the lack of sensitivity, which varies widely (20–80%) and so less in patients with paucibacillary TB including children, patients with extrapulmonary TB, or those who are human immunodeficiency virus (HIV) coinfected. The other disadvantage of microscopy is that it cannot differentiate between live and dead bacilli and hence cannot be used as a follow-up diagnostic test. 26

### Culture

Mycobacterial culture methods include conventional culture on solid agar (Lowenstein–Jensen media) and rapid culture methods include automated liquid culture system (e.g., mycobacterial growth indicator tube [MGIT] or BacT/ALERT MB). The BACTEC MGIT culture system is a fluorescent signaling system, and it provides an early recovery of *Mycobacterium* , that is, within 10 days as compared with 24 to 28 days by conventional culture methods, and drug susceptibility can be checked in a shorter time span. 25

### Molecular Assay

The diagnostic field of TB has seen the advances in form of new molecular tests often refer to as NAATs. These assays are based on the amplification of a targeted genetic region of the *M. tuberculosis* complex, by performing polymerase chain reaction. Along with the detection of TB, NAAT can perform drug susceptibility also and more quickly than conventional mycobacterial culture.

### Xpert MTB/RIF

Xpert MTB/RIF is a cartridge-based molecular assay that enables rapid detection of *M. tuberculosis* and simultaneous identification of rifampin resistance directly from clinical specimens of both pulmonary and extrapulmonary TBs. In 2017, Xpert Ultra (Cepheid) came as next-generation Xpert testing, as the initial TB diagnostic test for adults and children, irrespective of HIV status, over smear microscopy and culture. Under the current Revised National TB Control Program guidelines, it is recommended for diagnosis of drug-resistant TB (DR-TB) in presumptive DR-TB and upfront diagnosis of TB in key populations such as pediatric TB, extrapulmonary cases, and people living with HIV. The analytical limit is 131 CFU/mL, and the turnaround time is 2 to 3 hours, while Xpert Ultra has the lower limit of detection of 16 CFU/mL, hence further improving the sensitivity from 85 to 88%. 27

### Line Probe Assay

Line probe assays (LPAs) are a family of DNA strip-based tests that determine the drug resistance profile of an MTB strain through the pattern of binding of amplicons (DNA amplification products) to probes targeting the most common resistance-associated mutations to first- and second-line agents. LPAs are World Health Organization (WHO)-approved tests for the rapid detection of drug resistance to first- and second-line agents. These assays include GenoType MTBDRplus (Hain Lifesciences-Bruker, Nehren, Germany) and Nipro NTM_MDRTB II (Osaka, Japan). There are new-generation LPAs (e.g., GenoType MTBDRsl version 2.0; Hain Lifesciences-Bruker) with higher sensitivity and can detect mutations associated with fluoroquinolones and second-line injectable drugs—kanamycin, amikacin, and capreomycin and are recommended to guide multidrug-resistant (MDR) TB treatment initiation. 24 28

### Next-Generation Sequencing

Next-generation sequencing (NGS) is fast and considered a promising option for comprehensive drug-susceptibility testing for TB. NGS-based assays can provide detailed and accurate sequence information for whole genomes or multiple gene regions of interest. WHO has published guidance on the role of sequencing for detecting mutations associated with drug resistance in TB. 29

### TB Lipoaribomannan

TB lipoaribomannan (LAM) test detects lipopolysaccharide which is shed from mycobacterial cell walls in urine. It is primarily applicable to HIV/AIDS patients. Point-of-care strips for LAM is available on the market for use among the HIV-infected individuals. Importantly, the only approved antibody test for TB is the Alere LAM. 26

### Histopathological Features

In early-stage or active TB, there are pathologic findings of the caseous necrosis area and tuberculous granuloma in the adrenal gland because of the destruction of the cortex by tubercular bacilli. 15 Caseous necrosis, epithelioid granuloma, and Langhan's type of giant cells are characteristic of adrenal TB, as found in other forms of TB. Lack of granulomatous inflammation in active tubercular adrenal lesions may be found due to the local suppressive effect of steroids secreted in the adrenal cortex. Also, sometimes in adrenal insufficiency, a large proportion of the adrenal gland has been destroyed, and the local suppressive effect of steroids becomes minimal. Ziehl–Nielsen's staining should be performed for confirmation of TB. 25

### Latent Tuberculosis Infection

Latent tuberculosis infection (LTBI) is a state of persistent immune response to stimulation by *M. tuberculosis* antigens without evidence of clinically manifested active TB disease. In country like India, there is high burden of latency of TB. So, all the serological tests which detect immunoglobulin (Ig) G or IgM antibodies against mycobacteria and cellular immune response via skin tests such as tuberculin skin test and interferon-gamma release assays have no role in the diagnosis of active TB. Government has banned the use of serodiagnostic test kits for diagnosis of TB in India. 25

## Radiographic Features

CT scan is the main diagnostic imaging tool for adrenal TB. 16 The CT scan features of adrenal tuberculous with Addison's disease are bilateral mass-like enlargement, adrenal calcification, and peripheral rim enhancement with low attenuation in the center part of the adrenal glands. Peripheral rim enhancement with low attenuation in the center with calcification of the adrenal glands is a characteristic feature of adrenal TB. 14 16 CT scan can also tell about the early or active disease. As the duration of adrenal TB increases, the presence of peripheral rim enhancement decreases on progressive CT images. 30 CT-guided percutaneous fine-needle aspiration biopsy can be used to confirm the diagnosis of adrenal TB in suspected patients who present with adrenal gland mass seen incidentally using ultrasonography (USG) or CT scan of the abdomen. 31

MRI can also be used to evaluate pathological changes of adrenal TB. Zhang et al, in 2008, first reported MRI features in diagnosing adrenal TB. 32 MRI features are similar to CT scan such as bilateral involvement, mass-like enlargement, a hypointense or isointense signal of the central zone in the T2-weighted image, and peripheral rim enhancement. 32 Granuloma and caseous necrosis are more clearly evident in MRI, while calcification is more clearly visible in CT images. In chronic cases where the gland becomes atrophic, the glands would show low signal intensity on all MRI scans and have no increase in intensity on contrast-enhanced T1-weighted images. The presence of caseous necrosis and granuloma is, therefore, indicative of active TB, while the formation of fibrosis and calcification indicates lesions in a quiescent or chronic stage. 33 MRI should generally be considered superior to CT scan due to the fact that it is a noninvasive, high-resolution image, and radiation-free. MRI is also beneficial in the case of allergy to iodine-containing contrast. 32

## Treatment

The treatment of adrenal TB is the same as any other extrapulmonary tuberculous lesion. If it is associated with adrenal insufficiency, hormonal replacement is warranted. Conventional antitubercular therapy involving the use of a standard four-drug regimen remains the cornerstone of treatment. Treatment consists combination of isoniazid (5 mg/kg body weight [BW]/d), rifampicin (10 mg/kg BW/d), pyrazinamide (30 mg/kg BW/d), and ethambutol (20 mg/kg BW/d) generally for 3 to 6 months, subsequently isoniazid and rifampicin for 6 to 12 months. 5

For resistant cases or MDR, recurrence, relapse, or failure cases treatment for 18 to 24 months is recommended. 7

Treatment for adrenal insufficiency consists of lifelong hormone therapy with glucocorticoid and mineralocorticoid hormones. Different drugs, dosage, and monitoring techniques are summarized in Table 2 .

**Table 2:** Medication summary for adrenal insufficiency 34 35

Medications for the treatment of adrenal insufficiency			
Drugs	Dosage	Comments	Monitoring
Glucocorticoids			
Prednisolone	3–5 mg once a day	Use stress doses for illness, surgical procedures, and hospitalization	Symptoms of adrenal insufficiency to be monitored. Low to normal plasma adrenocorticotropic hormone levels indicate overreplacement
Hydrocortisone	15–25 mg divided into two or three doses per day	Use stress doses for illness, surgical procedures, and hospitalization	
Dexamethasone	0.5 mg once a day	Use intramuscular dose for emergencies and when unable to tolerate oral intake	
Mineralocorticoids			
Fludrocortisone	0.05–0.2 mg once a day	Dosage may need to increase by 0.2 mg per day in the summer because of salt loss from perspiration	Blood pressure, serum sodium and serum potassium levels
Androgens			
Dehydroepiandrosterone	25–50 mg once a day	Can improve mood and quality of life in women	Libido, mood, and sense of well-being

## Conclusion

Tuberculous Addison's disease has been decreased markedly in recent years due to high suspicion, anti-TB therapy, and better imaging modalities. The possibility of adrenal insufficiency should be considered when hyponatremia and labile blood pressure are observed in patients with active TB, or those having a history of TB. From different studies, it can be concluded that enlarged bilateral glands mean a recent and active infection requiring treatment, whereas small atrophic calcified glands are in favor of chronic and probably inactive infection. The diagnosis can be confirmed by USG-guided, or CT scan-guided biopsy with the histological study, which shows an epithelioid giant cellular granuloma with caseous necrosis with or without Langhan's type cell specific for TB. The mainstay of treatment is antitubercular drugs with steroids for adrenal insufficiency; however, in some cases, adrenalectomy may be required in resistant cases.
